# Molecular Mechanisms of Therapeutic Resistance in Hepatocellular Carcinoma

**DOI:** 10.3390/ijms27219690

**Published:** 2026-10-29

**Authors:** Karen Hoi Lam Li, Frances Sun, Samuel Chan, Roland Ching Yu Leung, Bryan Cho Wing Li, Jeffrey Sum Lung Wong, Tiffany Hoi Wing Fung, Jenny Wai Yan Lo, Alvin Hoi Wai Chan, Thomas Yau

**Affiliations:** 1 Department of Medicine, Queen Mary Hospital, The University of Hong Kong, Hong Kong, China; 2 Centre of Cancer Medicine, School of Clinical Medicine, The University of Hong Kong, Hong Kong, China

**Keywords:** hepatocellular carcinoma, HCC, therapeutic resistance, immune checkpoint inhibitor, cancer stem cells, tumour microenvironment, epigenetics, liquid biopsy, biomarker

## Abstract

Hepatocellular carcinoma (HCC) remains a major global health burden, and therapeutic resistance continues to limit the benefit from systemic treatment. Despite advances in systemic therapies, including immune checkpoint inhibitor-based combinations, antiangiogenic agents, and multitargeted tyrosine kinase inhibitors for patients with advanced HCC, primary non-response and acquired resistance are common. This review summarizes the mechanisms that underlie resistance to contemporary HCC therapies, including tumour heterogeneity, molecular pathway rewiring and crosstalk, tumour microenvironment and immune escape, epigenetic alterations, epithelial-to-mesenchymal transition, and cancer stem cell expansion. We discuss how these mechanisms limit the efficacy of current doublet and triplet regimens, and why biologically rational combinations do not consistently translate into clinical benefit. Emerging strategies to overcome resistance, including biomarker-enriched trial designs and liquid biopsy are also discussed. A deeper integration of mechanistic insights with translational biomarkers will be essential to improve treatment durability and clinical outcomes for patients with HCC.

## 1. Introduction

Hepatocellular carcinoma (HCC) is the most common form of liver cancer, accounting for approximately 90% of cases. Its highest incidence and mortality rates are observed in East Asia and Africa, but rising trends are also seen in Europe and America. Common risk factors include chronic hepatitis B or C virus (HBV or HCV, respectively) infection, alcohol consumption, and metabolic dysfunction-associated steatotic liver disease (MASLD). Telomerase activation is a major event in the pathogenesis of HCC in liver cirrhosis [1]. Cirrhosis of any cause represents the strongest risk factor, with HCC being the leading cause of mortality among patients with cirrhosis. HCC develops through genetic predisposition, the accumulation of genomic mutation, and epigenetic alterations influenced by both inherited and acquired mutations [1].

The combinations atezolizumab–bevacizumab, durvalumab–tremelimumab, and ipilimumab–nivolumab have emerged as the preferred first-line systemic therapies; all of them show overall survival (OS) benefits as compared with tyrosine kinase inhibitors, the first breakthrough treatment for advanced hepatocellular carcinoma [2]. Although combination immunotherapies have improved efficacy compared with monotherapies, suboptimal response rates and a median survival of under 2 years suggest that therapeutic resistance remains a substantial challenge [3]. Intrinsic and acquired resistance to systemic therapies in HCC limit their long-term benefits for patients and remains a significant unmet need. Patients who initially respond to systemic therapies may subsequently experience disease progression as acquired resistance emerges. Various strategies to ‘level up’ doublets by adding a biologically relevant third agent into triplet immunotherapies have faced setbacks [4,5]. A personalized, multidisciplinary, and multimodal approach appears to be the key to managing HCC [6]. Understanding the mechanisms of HCC resistance to systemic therapies helps to advance HCC treatment by enabling the development of the next wave of immunotherapies and targeted drugs with better pharmacological properties, the identification of predictive biomarkers and rational combination of systemic therapies.

**Figure 1:**
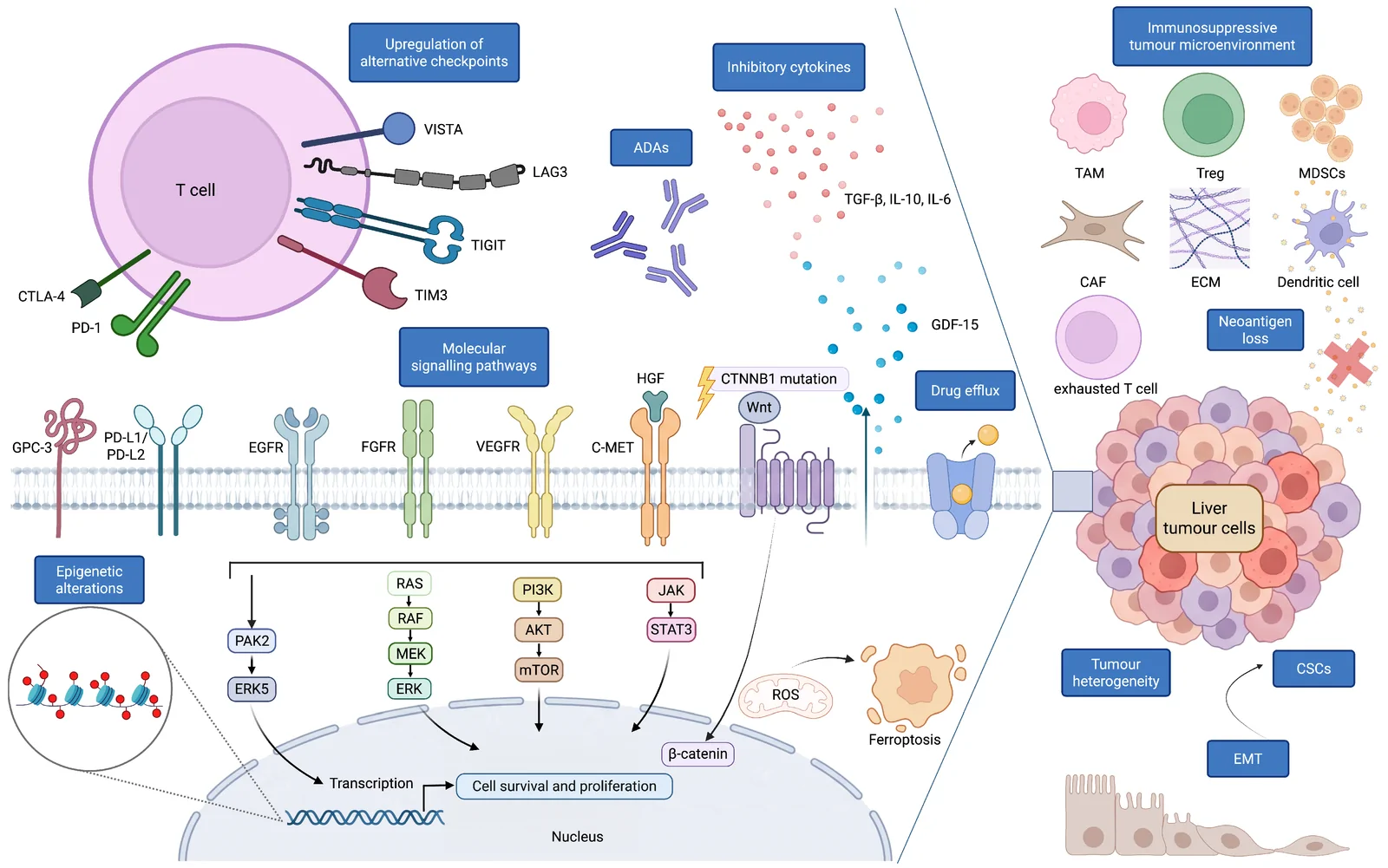
Mechanisms of therapeutic resistance in HCC. Created 2026. https://BioRender.com/0u50py0 (accessed on 16 October 2026). Therapeutic resistance in HCC is multifaceted, arising from convergence and crosstalks of multiple pathways. Tumour heterogeneity poses a fundamental barrier to the ‘one drug fits all’ approach. Abbreviations: V domain immunoglobulin suppressor of T-cell activation (VISTA), lymphocyte activation gene 3 (LAG-3), T-cell immunoreceptor with immunoglobulin and immunoreceptor tyrosine-based inhibitory motif (TIGIT), T-Cell Immunoglobulin and Mucin Domain 3 (TIM-3), cytotoxic T-lymphocyte-associated protein 4 (CTLA-4), programmed cell death protein-1 (PD-1), programmed cell death ligand 1 (PD-L1), programmed cell death ligand 2 (PD-L2), Glypican-3 (GPC3), epidermal growth factor receptor (EGFR), fibroblast growth factor receptor (FGFR), vascular endothelial growth factor receptor (VEGFR), hepatocyte growth factor (HGF), transforming growth factor-beta (TGF-β), Interleukins 10 and 6 (IL-10 and IL-6), growth differentiation factor 15 (GDF-15), tumour-associated macrophage (TAM), regulatory T cell (Treg), myeloid-derived suppressor cells (MDSCs), cancer-associated fibroblast (CAF), extracellular matrix (ECM), cancer stem cell (CSC), epithelial–mesenchymal transition (EMT).

HCC exhibits both intrinsic (or primary resistance) prior to exposure to systemic therapies and acquired resistance that develops through adaptive changes after drug exposure [7]. As HCC evolves, trunk mutations are present in all tumour cells, whereas private mutations may be present in only one part of the tumour. This leads to intratumuoral heterogeneity in HCC. Intertumour heterogeneity arises due to de novo independent carcinogenesis and intrahepatic metastasis. For example, for tumours with high immune intratumoural heterogeneity with vascular endothelial growth factor (VEGF) amplification, there is a predicted therapeutic benefit from combining an antiangiogenesis agent with a checkpoint inhibitor [8]. Heterogeneity in HCC is also attributed to cancer stem cells (CSCs) [9]. Tumour heterogeneity in HCC is a major contributor to the emergence of primary and secondary resistance to systemic therapies [1]. Three-dimensional (3D) organoids derived from HCC can recapitulate in vivo tumour heterogeneity and aid drug development and combinations [10]. The key mechanisms of therapeutic resistance in HCC are shown in Figure 1. Actionable targets in HCC and their therapeutic strategies are shown in Table 1.

## 2. Molecular Signalling Pathways

Genomic profiling of advanced HCC refractory to systemic therapies showed that TP53 (47.8%) and TERT promoter (46.4%) are the most frequently mutated genes; other notable altered gene include CTNNB1 (21.7%), RB1 (15.90%) and AXIN1 (10.1%). The main driver genes in HCC are often clonal, rendering them attractive therapeutic targets [1]. Oncogenic cascades in HCC are heterogeneous and do not function independently; rather, they exhibit extensive crosstalk. Although TP53 is the most frequently altered gene in HCC, it is difficult to target due to the lack of accessible pockets for drug binding and lacking enzyme activity, rendering catalytic inhibitors useless [49]. Two major molecular classes of HCC have been proposed—the proliferation class is associated with HBV infection, aggressive phenotypes, TP53 mutations and activation of pro-survival signalling pathways; the non-proliferation class is characterized by more differentiated tumours, related to HCV or alcohol, and often harbour CTNNB1 mutations [1]. Pre-existing or acquired alterations in cell signalling pathways may confer resistance to therapies targeting these pathways [50].

### 2.1. CTNNB1 Mutation and the Wnt/β-Catenin Pathway

Aberrant activation of the Wnt/β-catenin signalling pathway is observed in up to 50% of HCC cases, primarily because of a gain-of-function of CTNNB1. This is one of the factors associated with T-cell depletion and an immune-excluded ‘cold’ tumour microenvironment (TME), and it may be linked to primary resistance to immunotherapies [51,52]. Upregulation of cyclin-dependent kinase (CDK) 6 has been implicated in lenvatinib resistance in hepatocellular carcinoma through activating Wnt/β-catenin signalling activation, leading to the augmentation of cancer stem cells [53]. The intranuclear location of β-catenin makes drug inhibition challenging owing to its limited access, and, until recently, it was deemed undruggable. Monoclonal antibodies and small molecules targeting the Wnt/β catenin signalling pathway have been extensively studied, but their development is limited by on-target toxicity [54]. Recently, tegavivint, a first-in-class downstream Wnt/β-catenin inhibitor, demonstrated encouraging clinical activity in patients with pretreated advanced hepatocellular carcinoma in an early-phase trial [11]. This represents a potential novel therapeutic option warranting further development.

### 2.2. Epidermal Growth Factor Receptor (EGFR) Activation

EGFR is overexpressed in 68% of HCC cases and is associated with aggressive tumours and poor prognosis [55]. Patients who develop resistance to lenvatinib have been found to harbour EGFR and ERBB2 genetic alterations [56]. EGFR activation has been implicated in both primary and acquired resistance to tyrosine kinase inhibitors in HCC. Pharmacological inhibition of FGFR by lenvatinib leads to feedback activation of the EGFR–PAK2–ERK5 signalling axis, leading to primary unresponsiveness [57,58]. Acquired lenvatinib resistance in HCC is primarily driven by activation of the downstream STAT3-ABCB1 signalling pathway, which enhances drug efflux [58]. Similarly, EGFR bypass activation mediates acquired resistance to regorafenib via the RAS/RAF/ERK pathway [59]. Targeting EGFR alone is ineffective because of extensive crosstalk and upregulation of alternative pathways [60]. Combining lenvatinib or regorafenib with EGFR inhibitors, such as gefitinib, has demonstrated potent restorative antitumour efficacy in preclinical models [57,59]. However, sorafenib in combination with erlotinib did not improve overall survival in a phase III trial [15]. This exemplifies that mechanistic plausibility may not necessarily result in clinical success.

### 2.3. Fibroblast Growth Factor Receptor (FGFR) Signalling

Dysregulation of FGF/FGFR is associated with poor prognosis and the development of targeted therapies for HCC. Its mechanisms of resistance include crosstalk with other signalling pathways (e.g., MAPK, PIK3K/AKT/MTOR, STAT), promotion of angiogenesis, evasion of apoptosis, and EMT induction [61]. In a study using human HCC cell lines, activation of FGF19/FGFR4 signalling drove sorafenib resistance by inhibiting reactive oxygen species (ROS)-associated apoptosis in HCC previously treated with sorafenib [62]. FGFR1 overexpression drives lenvatinib resistance via the PI3K/AKT/mTOR and MAPK/ERK signalling pathways [17,63]. Ferroptosis is a unique, iron-dependent form of cell death driven by the accumulation of lethal lipid reactive oxygen species (ROS) [64]. Lenvatinib promotes ferroptosis in HCC by inhibiting FGFR4 [65]. Irpagratinib is an oral, highly selective FGFR4 inhibitor that, when combined with atezolizumab, showed promising antitumour activity in HCC patients with FGFR19 overexpression, whether treatment naïve or previously treated with immunotherapy [23]. While preclinical evidence showed that FGFR was mechanistically associated with HCC therapeutic resistance, more recent studies have demonstrated the clinical utility of FGFR inhibition in molecularly selected HCC.

### 2.4. Angiogenic Pathway

Angiogenesis is an important therapeutic target in HCC. Secreted vascular endothelial growth factor (VEGFs), upon binding to VEGF receptors (VEGFRs) expressed on endothelial cells, orchestrates endothelial cell proliferation, migration and survival in HCC [66]. Multitargeted tyrosine kinase inhibitors (TKIs), including sorafenib, lenvatinib, cabozantinib, regorafenib, and ramucirumab, a recombinant monoclonal antibody against VEGF-2—have improved survival in advanced HCC, yet the durability of benefit is usually limited following antiangiogenic therapy. Resistance to VEGF inhibition can be attributed to tumour adaptations that exploit alternative proangiogenic factors, such as FGF signalling, which undermines antiangiogenic effects [67]. Prolonged tumour angiogenesis blockade such as with sorafenib, can cause intratumoural hypoxia, allowing the selection of resistant clones adaptive to oxygen and nutrient depletion, typically exhibiting more aggressive phenotypes [68]. Vessel co-option is another resistance mechanism to sorafenib through which HCC tumour cells develop the ability to ‘hijack’ the existing vasculature in organs rather than relying on angiogenesis [69]. Activation of PI3K/AKT/mTOR and hypoxia-inducible factor-α (HIF-α) confers resistance to sorafenib [70]. Copanlisib, a PI3K inhibitor, was shown in a preclinical model that synergizes with sorafenib in treatment of advanced HCC [71]. Although the PI3K/AKT/mTOR signalling pathway is frequently dysregulated in HCC, clinical trials targeting this pathway have demonstrated limited efficacy [72]. Increased vascular endothelial growth factor release from lenvatinib-resistant HCC has been shown to enhance malignant tumour behaviours through its effects on tumour-associated macrophages (TAMs) in the tumour microenvironment [73]. In a mouse model with colorectal cancer, VEGF-A was shown to be a key driver of T-cell exhaustion by upregulating inhibitory checkpoints on CD8 T cells [74]. These findings may provide insight into why targeting angiogenesis alone in HCC is probably insufficient. Combining antiangiogenic agents such as anti-VEGF or multitargeted TKIs with immune checkpoint inhibitors addresses the interplay between angiogenesis and immune evasion, with a classic example being the combination of atezolizumab and bevacizumab in the IMbrave 150 trial [33].

### 2.5. HGF/c-MET Cascade

The HGF/c-MET pathway is another known mechanism of bypass in HCC. Aberrant activation of c-MET, a receptor for hepatocyte growth factor (HGF), drives HCC progression and stimulates key downstream signalling pathways, including PI3K/AKT/mTOR, Ras-Raf-MAPK and Wnt/β-catenin. These pathways promote tumour proliferation, invasion, metastasis, and the epithelial–mesenchymal transition (EMT) [75]. Treatment of HCC with sorafenib has been associated with increased MET expression in tumours, implying the role of MET in the emergence of sorafenib resistance [76]. Cabozantinib is a tyrosine kinase inhibitor with activity against multiple targets including c-MET, VEGFR1-3, RET, and the TAM kinase family (TYRO-3, AXL, and MER) [77]. The approval of cabozantinib was based on improved OS compared with placebo in patients previously treated with sorafenib in the phase III CELESTIAL trial [20]. Cabozantinib has been shown to modulate the tumour microenvironment, reduce immunosuppression and enhance CD8 T-cell-mediated cell death in various tumour models, leading to combination strategies of cabozantinib with immunotherapy with the aim of enhancing antitumour immunity [78]. The combination of cabozantinib plus atezolizumab was also tested in advanced HCC in the COSMIC-312 trial, which showed improved progression-free survival (PFS) and objective response rate without OS benefit compared to sorafenib [79]. This finding suggests the need for additional studies to identify patients who may benefit from this regimen. The triplet combination of nivolumab, ipilimumab and cabozantinib also showed a durable response with a manageable safety profile in sorafenib-pretreated patients with advanced HCC in phase I/II cohort 6 of the CheckMate 040 study [38].

## 3. Immune Tumour Microenvironment

### 3.1. Immunosuppressive Tumour Microenvironment

There is a constant, dynamic, and bi-directional interaction between liver cancer cells and the immune tumour microenvironment [8]. The liver maintains a tolerogenic microenvironment through the presence of specialized antigen-presenting cells and sinusoidal endothelial cells. Initially, tumour cells were targeted for immune destruction. The tumour microenvironment of HCC is dominated by immunosuppressive cells such as regulatory T cells (Tregs), myeloid-derived suppressor cells (MDSCs), and tumour-associated macrophages (TAMs), which secrete inhibitory factors such as IL-10 and TGF-β. The effector cells responsible for immunosurveillance and antitumour immunity include CD8 T cells, natural killer (NK) cells, some CD4 T cells and B cells [80]. CD8 T cells mediate cytotoxicity by releasing cytotoxic enzymes and cytokines [81]. Avoiding immune destruction is an emerging hallmark of cancer, and prolonged antigen exposure in chronic infections and cancers leads to T-cell exhaustion and dysfunction [82,83]. High levels of CD8 T cells and low levels of Tregs predict improved survival in patients with HCC [84]. The PD-1 and CTLA-4 immune checkpoint pathways suppress T-cell activation to preserve immune tolerance, thereby enabling tumour cells to evade immune surveillance. Additionally, upregulation of PD-1 and TIM3 can lead to T-cell exhaustion [81]. HCC is typically an ‘immune-cold tumour’. Despite the fact that approximately 25% of HCCs demonstrate a high or moderate level of tumour-infiltrating lymphocytes (TILs) within the tumour microenvironment, antitumour immunity is often insufficient due to the expansion of MDSCs and Tregs [85]. Apart from tumour cells, tumour-associated myeloid cells often overexpress PD-L1, which dampens immune surveillance through interaction with the PD-1 receptor on immune cells [86]. M2-type TAMs inhibit the migration of T cells activated by anti-PD-1 antibodies to tumour cells [87]. Cancer-associated fibroblasts (CAFs) secrete growth factors such as TGF-β, extracellular matrix proteins and angiogenic factors to create an immunosuppressive microenvironment and sustain stemness [88]. The release of exosomes and extracellular vesicles can modulate the tumour microenvironment distant from the primary tumour [89]. Anti-CTLA-4 antibodies can lead to Treg depletion; thus, combining CTLA-4 and PD-1 blockade is a common strategy used to overcome intrinsic tumour resistance. Nivolumab plus ipilimumab and the STRIDE regimen are approved for first-line treatment of unresectable HCC [29,32]. CTLA-4 primarily regulates T-cell activation during the priming stage in lymph nodes, whereas PD-1 inhibition predominantly enhances T-cell effector function at the tumour site [90]. The combination of immune checkpoint inhibitors with angiogenesis blockade can convert ‘cold’ tumours to ‘hot tumours’. Multitarget tyrosine kinase inhibitors can block the wnt/β-catenin, PI3K/AKT/mTOR, and CDK4/6 signalling pathways implicated in immune cell exclusion [80]. Lenvatinib plus anti-PD-1 has been shown to reduce Treg infiltration in experimental studies [91]. However, lenvatinib plus pembrolizumab did not improve OS or PFS as compared with lenvatinib alone in patients with advanced HCC [35]. While the same combination of lenvatinib plus pembrolizumab with TACE improves PFS compared with TACE alone in unresectable and non-metastatic HCC, longer follow-up is needed to determine if an OS benefit is present [92]. This shows that the translation from bench to bedside is complex. The addition of ipilimumab to atezolizumab plus bevacizumab was examined in the TRIPLET HCC trial. Treatment-related adverse events were frequent, leading to only around two-thirds of the patients being able to receive all four doses of ipilimumab, limiting the antitumour immunity response [4]. It has been proposed that a bispecific antibody may reduce toxicity as compared to two separate immune checkpoint inhibitors. In a phase II trial, the anti-PD-L1/CTLA-4 bispecific antibody KN046 combined with lenvatinib demonstrated promising efficacy in advanced HCC [93]. Novel strategies targeting Tregs have been explored in HCC [94]. Mogamulizumab, a monoclonal antibody targeting CCR4, induces antibody-dependent cellular cytotoxicity against CCR4-expressing regulatory T cells [95]. It is approved for the treatment of lymphoma and is being investigated in solid tumours. The development of Treg-directed strategies has been constrained by the risk of disrupting immunotolerance, leading to immune-related adverse events (irAE).

Forkhead box P3 regulatory T cells (Foxp3 Tregs) are central to hepatocarcinogenesis and immune escape, which display distinct aetiology-specific adaptations [94]. In chronic HBV and HCV infections, persistent viral antigen exposure is the primary driver of immune dysregulation. Viral hepatitis drives the expansion of highly suppressive Treg subsets—in particular, CCR4 and CD177 Tregs—and the exhaustion of CD8 T cells [94,96]. Tumour-associated macrophages (TAMs) secrete TGF-β/IL-10, while HCC tumour cells release CCL20 and CCL28, which promote Treg activity. This immunosuppressive environment impairs viral clearance and contributes to the progression of cirrhosis to HCC [97,98]. MASLD progresses to HCC via different mechanisms such as lipotoxicity, oxidative stress and inflammation [99]. In a mouse model, CD8 T cells in MASLD-related HCC demonstrated tissue-damaging potential, rendering PD1 or PD-L1 blockade ineffective [100]. In contrast, Treg impairment plays a permissive role in the immunopathogenesis of autoimmune hepatitis, resulting in excessive CD4 and CD8 T-cell immune responses [101]. Therefore, the controlled, nonresolving inflammation orchestrated by Tregs in viral hepatitis and MASLD appears highly permissive for tumour evolution, whereas the immune-mediated liver injury in autoimmune hepatitis is less oncogenic [102]. The molecular complexity and genetic heterogeneity of HCC help explain why some patients exhibit primary resistance to initial treatment [50].

### 3.2. Cytokines and Chemokines

Interleukins 1 and 6 (IL-1 and IL-6, respectively) promote liver carcinogenesis and play a role in drug resistance [50,103]. IL-6 is an inflammatory cytokine that is highly expressed in patients with HCC and is closely associated with HCC prognosis. It binds to its receptor (IL-6R) and triggers the activation of Janus kinase (JAK), which phosphorylates and activates signal transducer and activator of transcription 3 (STAT3), thereby promoting proliferation, survival, invasion and metastasis. The IL-6/STAT3 signal axis acts as a central mediator of HCC drug resistance by regulating drug-resistant proteins and activating other signalling pathways [104]. IL-6 also induces MDSCs, which promote immune invasion [105]. Anti-IL-6 and IL-6R antibodies such as Tocilizumab, JAK inhibitors and STAT3 inhibitors are supported by preclinical evidence [63]. A recently published retrospective study from the United States showed that patients with HCC who received their first dose of immune checkpoint inhibitor in the morning had reduced IL-6 levels and greater expansion of CD8 T cells after the first dose, with superior clinical outcomes, while those who received their first dose of immune checkpoint inhibitor in the afternoon had increased levels of IL-6 and less favourable T-cell expansion [106]. While randomized controlled trials are needed to confirm the morning dosing strategy for immunotherapies, it may represent a practical and inexpensive way to boost antitumour immunity. CXCR4 is a chemokine that is overexpressed in HCC but undetectable in normal liver tissue. It activates the PI3K/AKT/mTOR and MAPK/ERK pathways to promote therapeutic resistance by suppressing apoptosis [107]. Growth differentiation factor 15 (GDF-15) is a member of the TGF-β protein family [108]. It was initially discovered as a macrophage inhibitory cytokine crucial for foeto-maternal tolerance and was later found to play a key role in cancer cachexia [44]. By suppressing T-cell migration and function, GDF-15 creates an immunosuppressive tumour microenvironment that contributes to resistance to immunotherapy and tumour progression [108]; thus, it is an emerging therapeutic target in solid tumours, including HCC. Visugromab is a neutralizing anti-GDF-15 antibody that, in combination with nivolumab, showed deep responses in patients with HCC refractory to anti-PD1/anti-PD-L1 in the first-in-human phase I–IIa GDFATHER trial [44].

### 3.3. Neoantigen Loss

Tumours lacking neoantigens may evade T-cell recognition, thereby contributing to primary resistance to immune checkpoint inhibitors [109]. Under selective pressure from immune checkpoint inhibitors, tumour cells expressing neoantigens are preferentially eliminated by the immune system. Consequently, less immunogenic subpopulations depleted of neoantigen expand, a phenomenon known as immunoediting [110]. Downregulation of MHC-1 can also lead to impaired antigen presentation and recognition failure by CD8 T cells [111]. The hepatitis B virus impairs antigen presentation in HCC, impairing immunoproteasome function and reducing MHC class I expression [112]. Transarterial chemoembolization (TACE) stimulates neoantigen release and PD-L1 expression. The combination of TACE and immune checkpoint inhibitors is being investigated for intermediate-stage hepatocellular carcinoma, aimed at augmenting the immunogenicity of tumour cells [113]. Combination radiotherapy and immunotherapy for hepatocellular carcinoma has been shown to increase the volume and density of neoantigen presentation in preclinical studies [114]. Oncolytic virus therapy and neoantigen-based tumour vaccines are ways to elicit new immunogenicity in tumours depleted of neoantigens [115]. Antigen heterogeneity and loss have also been reported as one of the major challenges in the development of chimeric antigen receptor (CAR) T-cell therapy in HCC [116]. Combining CAR-T-cell therapy with immune checkpoint inhibitors, tyrosine kinase inhibitors or oncolytic viruses may improve the efficacy in HCC [116].

### 3.4. Upregulation of Alternative Immune Checkpoints

Beyond PD-1/PD-L1 and CTLA-4, the upregulation of alternative inhibitory checkpoints, such as lymphocyte activation gene 3 (LAG-3), T-Cell Immunoglobulin and Mucin Domain 3 (TIM-3), T-cell immunoreceptor with immunoglobulin and immunoreceptor tyrosine-based inhibitory motif (TIGIT), and V-domain immunoglobulin suppressor of T-cell Activation (VISTA) drives resistance to immunotherapy [117]. These represent one of the key mechanisms of acquired resistance to immunotherapies and may serve as therapeutic targets [118]. LAG-3 interacts with MHC II to evade immune cell activity and resist therapy-induced apoptosis [119]. Ex-vivo studies have shown that blocking LAG-3 increases the proliferation of CD4 and CD8 T cells [120]. Relatlimab is a first-in-class LAG-3-blocking antibody and was examined in the RELATIVITY-106 phase I/II trial [42]. Tebotelimab (PD-1/LAG-3 bispecific antibody) showed a manageable safety profile in patients with advanced hepatocellular carcinoma in a single-arm phase I/II trial [121]. TIM-3 leads to CD8 T-cell dysfunction and apoptosis and enhances immunosuppression mediated by Tregs [122]. TSR-022, a monoclonal antibody against TIM-3, was tested in a phase I trial [123]. TIGIT is expressed on several types of immune cells, including NK cells, Tregs, CD8 and CD4 T cells. TIGIT binds to CD155 to inhibit the signalling of the costimulatory CD226 receptor found on T and NK cells, leading to an exhausted phenotype [124]. Disappointingly, the addition of tiragolumab to atezolizumab and bevacizumab did not improve progression-free survival in advanced hepatocellular carcinoma in the phase III IMbrave152/SKYSCRAPER-14 study, despite promising data from the phase I/II MORPHEUS-LIVER trial [5,125]. The SKYSCRAPER-01 study examining anti-TIGIT in PD-L1-high advanced non-small-cell lung cancer also had negative results [124]. The failure of this mechanistically synergistic strategy may be due to pathway redundancy, CD226 downregulation, and a narrow biological window [126]. VISTA is a member of the B7 family of coreceptors for T-cell activation; it inhibits T-cell proliferation and suppresses immune response [127]. Targeting VISTA may have potential in halting therapeutic resistance.

## 4. Epigenetic Alterations in HCC

A comprehensive transcriptomic analysis of HCC identified several epigenetic genes such as EpiG5-T and EpiG1-L, that were upregulated in the immune-excluded phenotype [128]. Epigenetic alterations in HCC, including DNA methylation, histone modifications and noncoding RNAs (ncRNAs), play a significant role. Epigenetic alterations that cause silencing of gene expression and loss of mutation-associated antigens have been implicated in the mechanism of acquired drug resistance to immune checkpoint inhibitors through impairment of immunogenicity and immune recognition [129]. DNA methylation leads to reversible silencing of tumour suppression genes and the activation of oncogenes in HCC [130]. Its biomarkers can be detected in tissues, plasma or body fluids. DNA methylation contributes to therapeutic resistance in HCC by silencing apoptosis-related genes, promoting drug efflux through ATP-binding cassette (ABC) transporters, inducing hypermethylation of DNA repair genes, and altering the epigenetic regulation of cancer stem cells (CSCs) [130]. DNA methyltransferase (DNMT) inhibitors, such as decitabine and 5-azacytidine, used to treat acute myeloid leukaemia, may be potentially repurposed for the treatment of HCC [131]. The combination of guadecitabine, another DNMT inhibitor, with durvalumab is being tested in a phase Ib trial [48]. Histone modifications (acetylation, methylation, phosphorylation, etc.) are epigenetic changes that alter chromatin structure and gene expression without changing the DNA sequence. Histone acetyltransferase (HAT) and histone deacetylase (HDAC) regulate histone acetylation. Panobinostat, a histone deacetylase inhibitor, was evaluated in combination with sorafenib in patients with advanced HCC; however, the trial was terminated because of dose-limiting toxicity [132,133]. Zeste homolog 2 (EZH2), a key regulator of histone methylation, represents a potentially druggable therapeutic target [134]. EZH2 has also been shown to reduce ferroptosis. Tazemetostat is a first-in-class oral selective EZH2 inhibitor approved for metastatic or unresectable epithelioid sarcoma [135]. The addition of tazemetostat has been shown to increase the efficacy of sorafenib in vitro and in vivo, implying that combining EZH2 inhibitors with sorafenib could be a promising strategy to overcome drug resistance in HCC [136]. Most non-coding RNAs are not responsible for protein translation like typical coding RNA [131]. Long noncoding RNA (lncRNA) is a group of noncoding RNAs that have emerged as key players in HCC pathogenesis and therapeutic resistance. Lenvatinib-resistant HCC was shown to have upregulated IncRNA XIST. IncRNA XIST drives lenvatinib resistance by interacting with EZH2. Thus, the lncXIST/EZH2/NOD2/ERK axis might be an attractive therapeutic target [137]. OTX-2002-101 was the first-in-class mRNA therapeutic to downregulate MYC expression through epigenetic regulation [138].

Differential epigenetic changes associated with HBV/HCV- and MASLD-related HCC may lead to variable responses to therapeutic agents. Hepatitis B virus causes the upregulation of DNMTs, leading to hypermethylation and silencing of tumour suppressor genes, including IGFBP-3, RASSF1A, GSTP1, and CDKN2B. Chronic HCV infection causes global changes in H3K27ac, an activating histone. Upregulation of histone methyltransferase (e.g., SMYD2) and methylation reader (e.g., CBX1) in MASLD drives carcinogenesis. Epigenetic drugs alone or in combination with other systemic therapies remain a promising area that requires further research and development [139].

## 5. Epithelial-to-Mesenchymal Transition and Cancer Stem Cells

Epithelial–mesenchymal transition (EMT) is a process in which polarized epithelial cells transform into mesenchymal cells [140]. In fact, EMT is a continuum involving various continuous transitional states, rather than a binary switch. Cells in hybrid or partial EMT states possess both epithelial and mesenchymal properties with high plasticity and stemness potential [141]. Loss of E-cadherin expression is the hallmark of EMT [142]. EMT is mediated by transcription factors such as Twist, ZEB1, and Snail [143], and its development in HCC is driven by the tumour microenvironment, including CAFs, TAMs, hypoxia, extracellular matrix (ECM), and immune and inflammatory cells. EMT drives the overexpression of P-glycoprotein (P-gp), enhancing drug efflux and BCL-2 apoptotic proteins, leading to drug resistance [140]. It is regulated by several signalling pathways, including transforming growth factor-beta (TGF-β), Notch and Wnt/β-catenin. Galunisertib, a small-molecule selective inhibitor of the TGF-β receptor, in combination with sorafenib was tested in a phase II study for first-line treatment of HCC [12]. The PI3K inhibitor Idelalisib and LY294002 have been shown to suppress EMT in HCC by enhancing nuclear translocation of GSK-3β, thus decreasing Snail expression and consequently inhibiting PI3K/AKT signalling in HCC cell lines [144].

In 1997, Bonnet and Dick isolated the first cancer stem cells from a subset of CD34+CD38− acute myeloid leukaemia (AML) cells with the ability to initiate human AML in mice with severe combined immunodeficiency disease [145]. Liver cancer stem cells (CSCs) refer to a subset of tumour cells harbouring stem-cell-like characteristics for self-renewal and differentiation, which contributes to both primary and acquired therapeutic resistance [140,146]. CSCs in HCC primarily arise from hepatocytes, with emerging evidence that they may also be contributed by proliferating ductal cells. Well-established stemness markers in HCC include CD133, CD24, CD44, CD13 and EpCAM [147,148]. A variety of genetic and epigenetic factors that modulate various signalling pathways lead to cancer stemness [148]. Liver CSCs mediate therapeutic resistance through multiple mechanisms, including dormancy in the case of liver resection or transplantation, enhanced stemness and autophagy, plasticity and enhanced drug efflux. Liver CSCs interact with immune cells, including TAMs, neutrophils, NK cells and MDSCs, thereby promoting immune evasion [149]. The methods proposed to target liver CSCs include inhibition of cell surface proteins and immunotherapies, including cancer vaccine and CAR T cells to generate CSC-specific T cells [147].

EMT and CSC conversion are highly interconnected dynamic processes that play critical roles in disease progression and therapeutic resistance. EMT activation induces epigenetic changes that convert non-CSCs into CSCs. Cancer cells can transit back and forth between non-CSC and CSC states through EMT and its reverse process, mesenchymal-to-epithelial transition (MET). Transcription factors and signalling pathways implicated in EMT, such as TGF-β, Notch and Wnt/β-catenin also govern CSCs [150]. A study using HCC cell lines showed that TGF-β and CD147 form a self-reinforcing signalling network that promotes cancer stemness and plasticity in EMT in HCC [151]. Another study showed that HCC expressing keratin 19 exhibited CSC-like properties and was associated with EMT [152]. A better understanding of the dynamic relationship between EMT and CSCs could help in conquering therapeutic resistance in HCC.

## 6. Biomarker Development

### 6.1. Blood-Based Biomarkers

Alpha-foetoprotein (AFP) is a oncofoetal biomarker widely used in HCC. Patients with a baseline AFP of 400 ng/mL or greater showed improved overall survival benefits in the ramucirumab treatment arm of the phase III REACH-2 trial after progression on sorafenib [19]. An early AFP response at 3 weeks in combination with the albumin–bilirubin (ALBI) grade, was shown to correlate with radiological response and OS benefits [153]. The use of AFP as a biomarker in contemporary HCC treatment is controversial as the exact role of AFP in adults remains uncertain, making it an unattractive biomarker in current translational research [154].

Liquid biopsy was coined by Klaus Pantel et al. in 2010 [155]. Since then, considerable progress has been made. Apart from early detection, treatment monitoring and minimal residual disease detection, liquid biopsy is increasingly being used to detect therapeutic resistance [156]. Liquid biopsy of HCC conventionally involves circulating tumour cells (CTCs) and circulating tumour DNA (ctDNA) [157]. Research has explored the possibility of detecting microRNAs and exosomes as liquid biopsy biomarkers [158]. ctDNA analysis can track and detect the emergence of resistance-conferring mutations during therapy. Next-generation sequencing (NGS) can also be performed on ctDNA. CTCs can be analysed for protein expression and signalling pathway activation for phenotypic profiling [159]. For example, CTCs enriched with the pERK+/pAkt- phenotype are predictive of better sensitivity to sorafenib [160]. These help with longitudinal monitoring and the development of personalized medicine. Liquid biopsies and molecular markers in HCC remain investigational, and validation in large diverse cohorts is warranted [161].

Anti-drug antibody (ADA) positivity may be associated with early therapeutic resistance [162]. A prospective study of Korean patients reported that elevated levels of ADA against atezolizumab after treatment initiation may be associated with poorer clinical outcomes among those receiving atezolizumab plus bevacizumab. Patients with high ADA levels had reduced CD8 T-cell proliferation, interferon-γ, and tumour necrosis-α [163]. However, in the LEAP-002 study, only a small minority of patients developed treatment-emergent anti-drug-antibodies, without any correlation with pembrolizumab exposure [35].

### 6.2. Tissue Biomarkers

Glypican-3 (GPC-3) is overexpressed in 70–80% of HCC while undetectable in normal tissues, making it an attractive biomarker and therapeutic target. Further studies are needed to determine the level of GPC-3 expression required for GPC-3-directed therapies to be effective [164]. At the tissue level, elevated CAFs, M2-type TAMs and Tregs confer resistance to immunotherapy in HCC by contributing to an immunosuppressive tumour microenvironment [165]. A high intratumoural Foxp3 regulatory T-cell density and high Foxp3/CD4 ratio are associated with poorer overall survival and early recurrence in HCC [94]. Because HCC is often accompanied by cirrhosis, and thus, a prolonged prothrombin time, liver biopsy carries an increased risk of bleeding. A radiomics-based clinical model using gadolinium-ethoxybenzyl-diethylenetriamine (Gd-EOB-DTPA)-enhanced magnetic resonance imaging (MRI) was developed to predict the Immunoscore in HCC patients prior to initiation of systemic therapies. The Immunoscore measures CD3 and CD8 T-cell density in the tumour core and margin [166]. For HCC, a high Immunoscore was correlated with a lower recurrence rate and more prolonged recurrence-free survival [167,168]. Gd-EOB-DTPA MRI can also serve as a non-invasive imaging technique for WNT/β-catenin mutation, and thus, therapeutic resistance to immune checkpoint inhibitors [169]. The HCC Immunoscore may help to identify patients most likely to benefit from immunotherapy [166]. Inflammatory markers such as IL-6 are also being studied as predictive biomarkers for HCC [170].

### 6.3. Biomarkers Associated with the Host

HCC aetiology has been an area of interest as a biomarker for response or resistance to immunotherapy. A systematic review and meta-analysis of randomized controlled trials 2002–2020 showed there that immune checkpoint inhibitors may be more effective for HCC with viral aetiologies; however, these trials were not stratified by aetiology, and more prospective data are needed [171]. Future clinical trials should consider prespecified subgroup analyses of viral and non-viral aetiologies to better understand whether HCC treatment should be aetiology-based. The gut microbiome has also been shown to modulate response to immune checkpoint inhibitors in patients with HCC and may predict treatment outcomes. A study showed that non-responders to immunotherapy have several gut microbiome characteristics, including increased Proteobacteria, especially Escherichia coli and decreased beneficial gut species [172].

### 6.4. Incorporation of Biomarkers into Clinical Trials

When appropriate from scientific and ethical standpoints, the use of paired tissue sampling in clinical trials can assist with biomarker discovery and drug resistance [173]. Biomarker testing is being increasingly integrated into contemporary HCC trials. For patients treated with neoadjuvant nivolumab plus ipilimumab followed by surgery, immunotherapy-induced tertiary lymphoid structure formation (TLS) and T-cell exhaustion appear to be promising biomarkers for predicting response to immunotherapy [174]. Although the combination of lenvatinib and nivolumab in the IKF-t006/IMMUNIB phase II study failed to meet the primary endpoint of improving ORR, the study analysed plasma epigenomic and tissue transcriptomic profiling to identify potential responders [36].

As opposed to the conventional all-comers strategy, biomarker enrichment trials only enrol patients with the prespecified biomarker and are randomized to receive either the study arm or the control arm. The phase Ib/II MORPHEUS-Liver trial exemplifies innovative trial design in HCC. It is a randomized umbrella trial that examines multiple study arms against a shared control arm. It also allows treatment arms to be added or discontinued as evidence accumulates, enabling the investigators to evaluate promising combinations and stop ineffective regimens [175]. Basket trials enrol patients with the same molecular target across different cancer types, including HCC [176]. Master observation trials prospectively collect biomarker and clinical data from a broad patient population, enabling biomarker discovery and therapeutic development [176]. Innovative trial designs will likely play a crucial role as we advance towards biomarker-driven therapies for HCC [175].

## 7. Conclusions and Future Perspectives

Over the past two decades, systemic therapies for advanced hepatocellular carcinoma have evolved dramatically from single-agent tyrosine kinase inhibition to potent combination regimens with antiangiogenic agents and dual immune checkpoint blockade. Despite these notable and groundbreaking therapeutic advances, primary non-responsiveness and acquired disease progression remain formidable barriers to durable disease control. As highlighted in this review, therapeutic resistance in HCC is not driven by an isolated mechanism but rather arises from convergence and coordinated interplay between tumour-intrinsic adaptations, including CTNNB1 mutations, bypass signalling and the immune tumour microenvironment, epigenetic alterations, epithelial-to-mesenchymal transition, and cancer stem cell expansion. This enables tumour cells to bypass therapeutic pressure and sustain proliferation, angiogenesis and survival. As a result of tumour heterogeneity at the molecular level and the limitations of preclinical models, biologically rational combinations in preclinical models show inconsistent clinical benefits when applied without adequate patient selection. Comprehensive molecular reclassification of HCC by using multiomic profiling may guide personalized therapeutic decisions in HCC for overcoming therapeutic resistance [161,176]. Novel drugs or combination strategics to overcome therapeutic resistance should have acceptable and manageable toxicity profiles.

Surmounting these redundant resistance programs necessitates moving towards adaptive and biomarker-enriched trials, multidisciplinary approaches in combining locoregional therapies with systemic treatment and novel systemic therapies with better pharmacological properties. Translating mechanistic insights into clinical efficacy is not easy given the complex network of therapeutic resistance underlying HCC. This will rely on non-invasive liquid biopsies and functional radiomics, enabling real-time monitoring of clonal evolution and immunoediting. Furthermore, refining therapeutic sequencing requires integrating multi-parameter biomarker frameworks, encompassing aetiology-specific biology, inflammatory indices, and spatial immunophenotyping to accurately stratify patients, initially in drug trials and ultimately in everyday treatment decisions.

## Figures and Tables

**Table 1:** List of actionable targets and potential therapeutic strategies in advanced HCC.

Target	Therapy	Phase	Line	Key Outcomes
Wnt/β-catenin	tegavivint (TBL1 inhibitor)	I/II	≥1	≤3 previous lines of treatment: PFS 8 months, ORR 22% [11]
TGF-β	galunisertib (TGF-β1 inhibitor) + sorafenib (VEGFR/RAF inhibitor)	II	1	OS 18.8 months, TTP 4.1 months [12]
	livmoniplimab (antibody against TGF-β1 activator) + budigalimab (anti-PD-1)	II/III	1	Pending [13]
JAK	itacitinib (JAK1 inhibitor)	Ib	≥2	OS 7.4 months, PFS 3.5 months [14]
EGFR	erlotinib (EGFR inhibitor) + sorafenib (VEGFR/RAF inhibitor)	III	1	vs. placebo + sorafenib OS 9.5 vs. 8.5 months, HR 0.929, *p* = 0.408 [15]
VEGF	sorafenib (VEGFR/RAF inhibitor)	III	1	vs. placebo OS 10.7 vs. 7.9, HR 0.69, *p* < 0.001 [16]
	lenvatinib (VEGFR 1, 2, 3 inhibitor)	III	1	vs. sorafenib OS 13.6 vs. 12.3 months, HR 0.92 (non-inferior to sorafenib) [17]
	regorafenib (VEGFR2/PDGFR inhibitor)	III	2	vs. placebo; progression on sorafenib OS 10.6 vs. 7.8 months, HR 0.63, *p* < 0.0001 [18]
	ramucirumab (VEGF2 inhibitor)	III	2	vs. placebo, AFP ≥ 400 ng/mL OS 8.5 vs. 7.3 months, HR 0.710, *p* = 0.0199 [19]
MET	cabozantinib (VEGFR1, 2, and 3, MET, and AXL inhibitor)	III	≥2	vs. placebo OS 10.2 vs. 8.0 months, HR 0.76, *p* = 0.005 [20]
	tepotinib (MET inhibitor)	Ib/II	1	vs. sorafenib, MET overexpression TTP 2.7 vs. 1.4 months, HR 0.42, *p* = 0.0043 [21]
FGFR	fisogatinib (FGFR4 inhibitor)	I	≥1	FGF 19-positive: ORR 17% [22]
	irpagratinib (FGFR4 inhibitor) + atezolizumab (anti-PD-L1)	II	≥1	FGF 19-positive: mPFS 7 months, DOR not reached, ORR 51.7% [23]
RAS	refametinib (MEK inhibitor) + sorafenib (VEGFR/RAF inhibitor)	II	1	RAS mutation: mOS 12.7 months, PFS 1.5 months, ORR 6.3% [24]
PI3K/AKT/mTOR	siroimus (mTOR inhibitor)	II	1	OS 26.4 weeks, PFS 15.3 weeks, ORR 8% [25]
	temsirolimus (mTOR inhibitor) + sorafenib	II	1	OS 8.8 months, TTP 3.7 months [26]
	everolimus (mTOR inhibitor)	III	2	vs. placebo, progression on sorafenib OS 7.6 vs. 7.3 months, HR 1.05, *p* = 0.68 [27]
PD-(L)1	nivolumab (anti-PD1)	III	1	vs. sorafenib OS 16.4 vs. 14.7 months, HR 0.85, *p* = 0.075 [28]
	durvalumab (anti-PD-L1)	III	1	OS 16.56 months, HR 0.86 (non-inferior to sorafenib) [29]
	pembrolizumab (anti-PD1)	III	2	vs. BSC OS 13.8 vs. 10.6 months, HR 0.781, *p* = 0.0238 [30]
	avelumab (anti-PD-L1)	II	2	OS 14.2 months, TTP 4.4 months, ORR 10% [31]
PD-(L)1/CTLA-4	nivolumab + ipilimumab (anti-CTLA-4)	III	1	vs. sorafenib or lenvatinib OS 23.7 vs. 20.6 months, HR 0.79, *p* = 0.018
	durvalumab + tremelimumab (anti-CTLA-4)	III	1	vs. sorafenib [32] OS 16.43 vs. 13.77, HR 0.78, *p* = 0.0035 [29]
PD-(L)1/VEGF	atezolizumab + bevacizumab (anti-VEGF-A)	III	1	vs. sorafenib OS 19.2 vs. 13.4 months, HR 0.66, *p* < 0.001 [33]
	atezolizumab + cabozantinib	III	1	vs. sorafenib OS 16.5 vs. 15.5, HR 0.98, *p* = 0.87 [34]
	pembrolizumab + lenvatinib	III	1	vs. lenvatinib OS 21.2 vs. 19.0, HR 0.84, *p* = 0.023 [35]
	nivolumab + lenvatinib	II	1	OS 26.6 months, PFS 9 months, ORR 32% [36]
	atezolizumab + lenvatinib or sorafenib	III	2	vs. lenvatinib or sorafenib alone, progression atezolizumab and bevacizumab OS 14.6 vs. 12.5 months, HR 0.88, *p* = 0.2115 [37]
PD-(L)1/CTLA-4/VEGF	nivolumab + cabozantinib + ipilimumab	I/II	1/2	OS 22.1 months, PFS 4.3 months, ORR 29% [38]
	atezolizumab + bevacizumab + ipilimumab	II	1	ORR 30% [4]
	durvalumab + tremelimumab + bevacizumab	II	1	Pending [39]
TIGIT	tiragolumab (anti-TIGIT) + atezolizumab + bevacizumab	III	1	vs. placebo + atezolizumab + bevacizumab mPFS 8.2 vs. 8.3 months, HR 0.97 OS HR 0.94 [5]
	domvanalimab (anti-TIGIT) + zimberelimab (anti-PD-1)	II	≥2	PFS 4.4 months, ORR 17.2% [40]
TIM3	cobolimab (anti-TIM-3) + dostarlimab (anti-PD-1)	II	1	OS 27.3 months, PFS 11.0 months, DOR 14.8 months, ORR 37.1% [41]
LAG-3	nivolumab + relatlimab (anti-LAG-3) + bevacizumab	I/II	1	Pending [42]
	nivolumab + relatlimab	II	2	Pending [43]
GDF-15	visugromab (anti-GDF-15) + nivolumab	I/IIa	≥1	ORR 15.8% [44]
GPC3	AZD9793 (CD8-guided T-cell engager)	I/II	≥2	Pending [45]
Epigenetics	vorinostat (histone deacetylase inhibitor) + sorafenib	I	≥1	SD 77%, DOR 4.7 months [46]
	resminostat (histone deacetylase inhibitor) + sorafenib	I/II	1	OS 14.1 months, TTP 2.8 months [47]
	guadecitabine (DNMT inhibitor) and durvalumab	Ib	≥1	Pending [48]

Abbreviations: time to progression (TTP), overall response rate (ORR), hazard ratio (HR), stable disease (SD), duration of response (DOR).

## Data Availability

The original contributions presented in this study are included in the article. Further inquiries can be directed to the corresponding author.

## Abbreviations

The following abbreviations are used in this manuscript:

AML	acute myeloid leukaemia
ALBI	albumin–bilirubin
AFP	alpha fetoprotein
ADA	anti-drug antibody
ABC	ATP-binding cassette
CSC	cancer stem cell
CAF	cancer-associated fibroblast
CAR	chimeric antigen receptor
CTC	circulating tumour cell
ctDNA	circulating tumour DNA
CDK	cyclin-dependent kinase
CTLA-4	cytotoxic T-lymphocyte-associated protein 4
DNMT	DNA methyltransferase
DOR	duration of response
EGFR	epidermal growth factor receptor
EMT	epithelial–mesenchymal transition
ECM	extracellular matrix
EZH2	enhancer of zeste homolog 2
FGFR	fibroblast growth factor receptor
Foxp3+ Tregs	forkhead box P3 regulatory T cells
Gd-EOB-DTPA	gadolinium-ethoxybenzyl-diethylenetriamine
GPC-3	Glypican-3
HR	hazard ratio
HBV	hepatitis B virus
HCV	hepatitis C virus
HCC	hepatocellular carcinoma
HGF	hepatocyte growth factor
HAT	histone acetyltransferase
HDAC	histone deacetylase
HIF-α	hypoxia-inducible factor-α
IL-1	interleukin-1
IL-6	interleukin-6
JAK	Janus kinase
lncRNA	long noncoding RNA
LAG 3	lymphocyte activation gene 3
MET	mesenchymal-to-epithelial transition
MDSC	myeloid-derived suppressor cell
NK	natural killer
NGS	next-generation sequencing
ncRNA	noncoding RNA
OATP1B3	organic anion transporter
ORR	overall response rate
OS	overall survival
P-gp	P-glycoprotein
PD-L1	programmed cell death ligand 1
PD-1	programmed cell death protein-1
PFS	progression-free survival
ROS	reactive oxygen species
Treg	regulatory T cell
STAT3	signal transducer and activator of transcription 3
SD	stable disease
TIM-3	T-Cell Immunoglobulin and Mucin Domain 3
TIGIT	T-cell immunoreceptor with immunoglobulin and immunoreceptor tyrosine-based inhibitory motif
TLS	tertiary lymphoid structure formation
3D	three-dimensional
TACE	transarterial chemoembolization
TGF-β	transforming growth factor-beta
TTP	time to progression
TME	tumour microenvironment
TAM	tumour-associated macrophage
TIL	tumour-infiltrating lymphocyte
TKI	tyrosine kinase inhibitor
VISTA	V domain immunoglobulin suppressor of T-cell activation
VEGF	vascular endothelial growth factor
VEGFR	vascular endothelial growth factor receptor
